# Single-cell quantitative expression of nicotinic acetylcholine receptor mRNA in rat hippocampal interneurons

**DOI:** 10.1371/journal.pone.0301592

**Published:** 2024-04-18

**Authors:** Doris C. Jackson, Richard M. Burgon, Spencer Thompson, Sterling N. Sudweeks

**Affiliations:** Department of Cell Biology and Physiology, College of Life Sciences, Brigham Young University, Provo, Utah, United States of America; Weizmann Institute of Science, ISRAEL

## Abstract

Hippocampal interneurons are a very diverse population of cells. Using single-cell quantitative PCR to analyze rat CA1 hippocampal interneurons, we quantified neuronal nicotinic acetylcholine receptor (nAChR) mRNA subunit expression and detailed possible nAChR subtype combinations for the α2, α3, α4, α5, α7, β2, β3, and β4 subunits. We also compared the expression detected in the *stratum oriens* and the *stratum radiatum* hippocampal layers. We show that the majority of interneurons in the CA1 of the rat hippocampus contain detectable levels of nAChR subunit mRNA. Our results highlight the complexity of the CA1 nAChR population. Interestingly, the α3 nAChR subunit is one of the highest expressed subunit mRNAs in this population, while the α4 is one of the least likely subunits to be detected in CA1 interneurons. The β2 nAChR subunit is the highest expressed beta subunit mRNA in these cells. In addition, Pearson’s correlation coefficient values are calculated to identify significant differences between the nAChR subunit combinations expressed in the CA1 *stratum oriens* and the *stratum radiatum*. Statistical analysis also indicates that there are likely over 100 different nAChR subunit mRNA combinations expressed in rat CA1 interneurons. These results provide a valid avenue for identifying nAChR subtype targets that may be effective hippocampus-specific pharmacological targets.

## Introduction

Understanding the scope of expression of nicotinic acetylcholine receptors (nAChRs) within hippocampal interneurons would lend great insight for cognitive therapies used in Alzheimer’s Disease [1, 2], Attention-Deficit Hyperactivity Disorder [3], Autism Spectrum Disorder [4], and Post-Traumatic Stress Disorder [5]. The GABAergic interneurons are controlled by nAChRs found on their dendrites, soma, and on the axon terminals [6]. These GABAergic interneurons may be few in number, but they play an important role in regulating the synchronous firing of the much more numerous hippocampal pyramidal neurons [7, 8].

Within the hippocampus, whole cell electrophysiological recordings of CA1 interneurons show a wide range of kinetics in response to acetylcholine. The pentameric nAChR appears to be assembled by various combinations of the α2-α5, α7, and β2-β4 nAChR subunits, based on our single-cell qPCR mRNA detection. The nAChRs in hippocampal interneurons are in a key position to modulate cognitive functions because interneurons play a major role in coordinating hippocampal activity, as reviewed in [9, 10]. Accordingly, nAChRs have been implicated by many studies as having a major role in influencing cognition [11–14].

Variations of nAChR subtypes, composed of different pentameric combinations of subunits, within the hippocampus have previously been shown to have a wide range of nicotinic receptor kinetics [15] and enable plasticity in interneuron function [16, 17]. Of the neuronal nAChR subtypes, α7 containing receptors have been one of the most extensively studied. Use of subunit-specific antagonists to α7 homomeric receptors (either α-bungarotoxin or methyllycaconitine) has provided an important tool in isolating contributions of this subunit in receptor kinetics, physiology, and disease. There are many functional roles attributed to the α7 nAChR (within and outside of the hippocampus) including depression, cognition, gastric cancer, and memory [17–19].

The limited number of highly subunit-specific antagonists for other subtypes of the nAChR has made for slower progress in characterizing the roles of other subunit combinations to the same extent as the α7 subtype. Until more subunit-specific agents are discovered, other means for characterizing the individual nAChR subunits must also be utilized. On-going studies to characterize the extent of nAChR subunit co-expression within individual neurons may identify trends shared by functionally distinct networks or subpopulations of neurons [20]. In addition, the identification of co-expression trends between nAChR subunits and other neurochemical markers may facilitate more comprehensive characterization of the nAChR and of neuronal subpopulations. Therefore, we sought to differentiate and classify hippocampal interneuron expression of neuronal nAChR subunit mRNA through single-cell quantitative PCR.

When analyzing gene expression in the brain, various techniques like immunohistochemistry, in situ hybridization, and single-cell RNA expression are used [21, 22]. Each has it’s own set of advantages and disadvantages. Immunohistochemistry allows for the visualization of protein localization within tissues. This excels at locating populations of cells expressing a common protein of interest. However, its resolution isn’t as fine-grained, potentially missing rare cell types or protein expression nuances within a cell. Likewise in situ hybridization is excellent for analyzing the mRNA expression of groups of cells, but again the resolution of single cells can be more limited, especially with minority populations of cells that are interspersed among many other cell types, as is the case with hippocampal interneurons.

Single-cell sequencing, on the other hand, shines a spotlight on individual cells. This provides unparalleled resolution, including detailed quantitative analysis showing high resolution of quantitative comparisons of various mRNAs to each other using qPCR. This technique can be especially helpful in analyzing cell populations composed of relatively low numbers of diverse cell types (like hippocampal interneurons) that are anatomically interspersed with other more numerous cell types. However, there is always the caveat that mRNA expression doesn’t always directly translate to protein levels.

**Fig 1:**
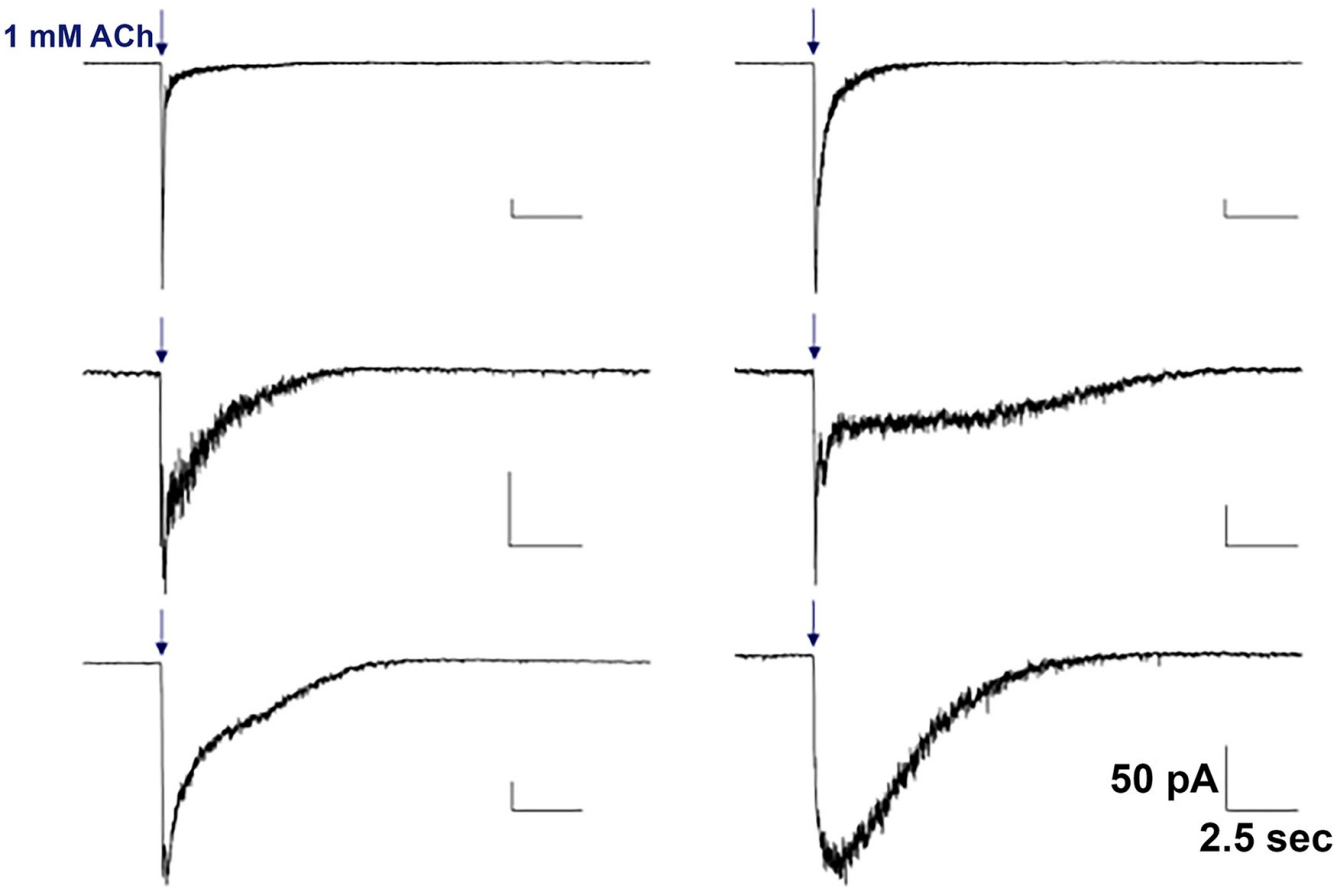
Electrophysiological recordings depicting the kinetic diversity of the nAChR population in rat hippocampal interneurons. Each trace was elicited using a 1 second, 1 mM ACh application during patch clamp experiments (timing indicated by downward arrows). Whole-cell recordings were made on the interneuron cell bodies. Scale bars are included for each recording (vertical bars = 50 pA, horizontal bars = 2.5 s). Each recording has been scaled to the maximum peak size to allow for easier visual comparison of the recording kinetics (relative rise-times, decay times, desensitization, etc.).

**Fig 2:**
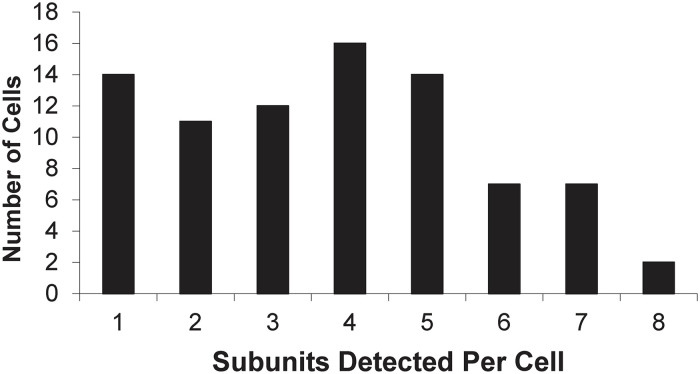
nAChR subunit expression by cell. A histogram reporting the number of nAChR mRNA subunits detected in each individual interneuron using qPCR (n = 93 neurons examined, 83 were positive for nAChR mRNA). The average number of subunits per cell was 3.4 (± 0.2 S.E.M.). This mean is calculated from all 93 neurons examined, i.e., including the 10 cells with no detectable subunit mRNA expression. A simplified summary of expression is given in Table 6, which reports the proportion of cells expressing each of the nAChR mRNA subunits examined (n = 93). Somewhat surprisingly, since it is traditionally associated with expression in the peripheral nervous system–not in the central nervous system, the α3 subunit was one of the most common nAChR subunits expressed in this population, found in 53.8% of the interneurons examined. Also unexpected was that the α4 subunit had the lowest expression of all the nAChR subunits analyzed (26.9%), since it is one of the most widely expressed nAChR subunits in the CNS overall. Fig 3 shows the relative expression of each subunit as histograms normalized to the median expression level for all of the nAChR subunits detected. This figure highlights both the high level of expression and the larger number of cells that express the α3, α5, β2, and β4 mRNA subunits, where those histograms are all skewed to the right (indicating higher expression levels). The α4 histogram reiterates the small number of cells expressing α4 and also indicates that the relative expression levels are low when compared to the average fold expression.

**Fig 3:**
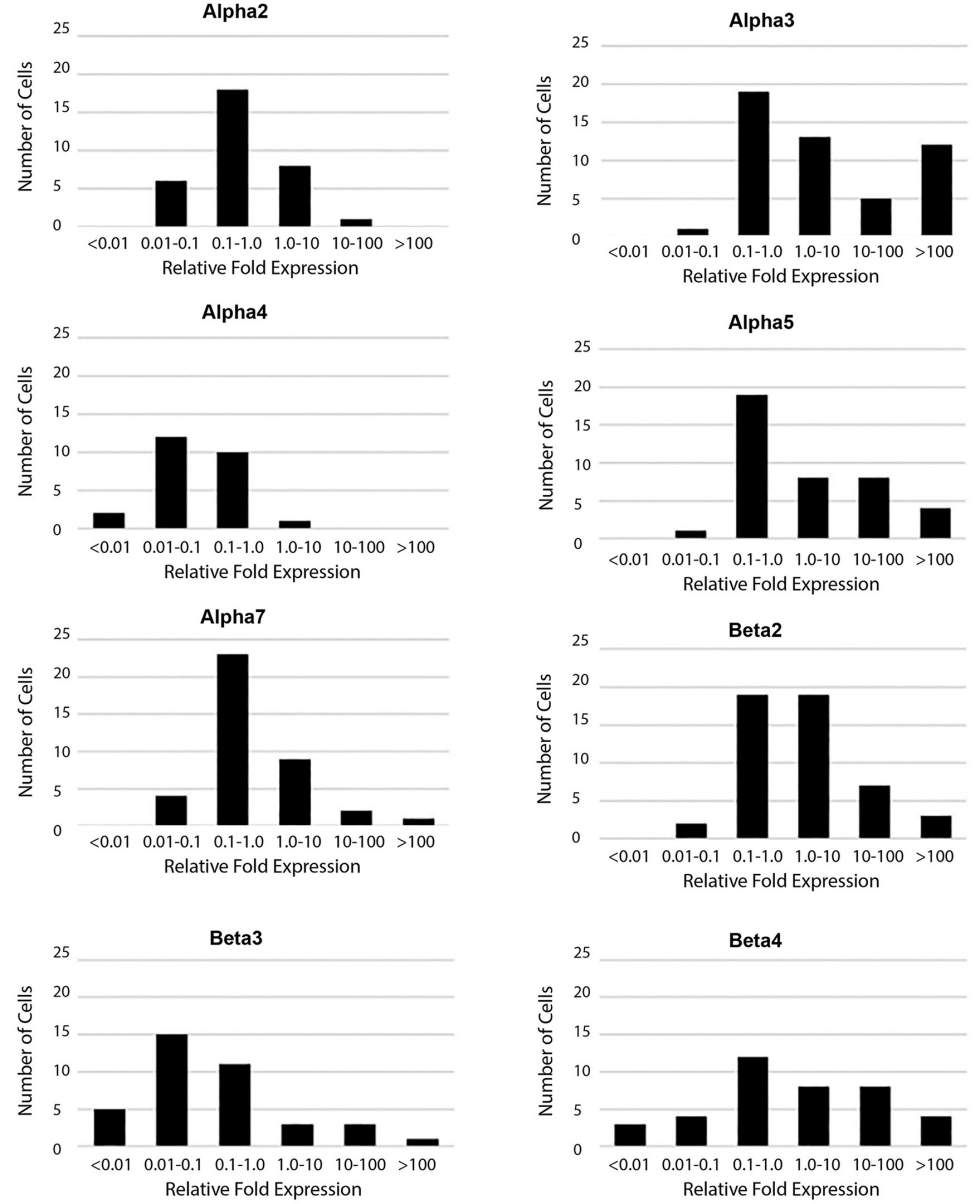
nAChR subunit expression by subunit. The mRNA expression levels of each nAChR subunit are indicated by histograms. Relative expression levels were binned on a logarithmic scale (X-axis). Each bin represented a 10 fold difference in the level of expression with 1 being the median fold expression level detected when looking at all of the nAChR subunit mRNAs. The total number of cells varies by subunit since it is based on the number of cells expressing that particular subunit. The highest possible n value is 83 since 83 of the 93 cells had detectable nAChR subunit mRNA.

In hippocampal interneurons from the *stratum oriens* and the *stratum radiatum*, we see diversity in both the single-cell activity of the proteins–as shown by the various kinetic responses shown at the single-cell level by whole-cell voltage-clamp electrophysiology of neuronal nAChRs (see Fig 1) and in the single-cell mRNA expression of neuronal nAChRs (Figs 2 and 3) detected through single-cell reverse-transcription qPCR. This indicates that both techniques are in agreement with the idea that many neuronal nAChR subunit types are expressed by hippocampal interneurons at both the protein and mRNA levels.

## Methods

### Slice preparation

IUCAC approval was obtained for all animal experiments. Pregnant female Wistar rats (Wistar IGS strain code 003) were obtained from Charles River Labs. 8 to 35 days old male and female rat pups were used to make the coronal hippocampal brain slices in these experiments. The following procedures were taken to ensure a minimization of animal suffering. Rats were anesthetized using isoflurane co-delivered with oxygen (2–4 liters per minute) from an AM Bickford Vapomatic 61020 veterinary anesthesia machine purchased from Kent Scientific. To ensure that an adequate depth of anesthesia was obtained, the loss of the withdrawal reflex on the rats when tugging lightly on one of the rear limbs was verified. Adult female mice were sacrificed using CO_2_ delivery in a chamber, followed by decapitation. Following anesthesia, rat pups were quickly decapitated using shears. The brains were then quickly removed and cooled by placing in ice-cold artificial cerebrospinal fluid (ACSF) oxygenated with 95% O_2_ + 5% CO_2_. To obtain the interneurons, coronal brain slices (either 300 or 350 μm thick) were made using a Vibratome 1000-Plus. The slices were also cut in ice-cold oxygenated (95% O_2_, 5% CO_2_) ACSF and placed in room-temperature oxygenated ACSF for at least 30 minutes prior to placing in microscope recording chamber.

### Cytoplasm aspiration

An upright microscope with infrared light was used to identify individual hippocampal interneurons from the Amun’s horn (CA1). Interneurons in the *stratum oriens* and *stratum radiatum* were visually identified as neurons outside the pyramidal cell layer. Electrophysiological recordings taken from a sample of these neurons verified that they showed spontaneous action potential activity, and responded to the application of acetylcholine with inward currents typical of nicotinic acetylcholine receptors (see Fig 1). Interneurons were then aspirated into a standard whole-cell patch-clamp pipette containing 5 μL Intracellular Fluid as described previously [15].

### Electrophysiology

A whole-cell recording configuration of the interneuron was obtained in voltage-clamp mode prior to cytoplasm aspiration. Interneuron membrane potentials were held at -70mV. Where recordings were performed, responses were induced by the gravity flow application of 1 mM ACh for 1 second, using an Axon 200. Whole-cell recordings were filtered at 1 kHz, and sampled at 100 Hz, using the pCLAMP Clampex software (version 7.01.31, Axon Instruments). Acetylcholine (ACh) was applied using an electronically triggered valve (General Valve Co., Fairfield, NJ, USA) connected to a synthetic quartz tube (inner diameter of 320 μm, Polymicro Technologies Inc., Phoenix, AZ, USA) placed approximately 90–120 μm from the cell body. The flow rate for the ACh application from the gravity flow system was 250 μl min. A washout period of at least 3 min was included between subsequent recordings.

### Primers and probes

The primers and probes were designed using either Vector NTI version 7.0 (Invitrogen) or Primer Express version 2.0 (ABI Prism) software. The primer and probe sequences used are listed in Tables 1 & 2.

**Table 1:** Amplicon lengths and oligo sequences of subunit probes.

Real-time PCR probes	Amplicon Length	Probe
nAChR β2 rat	77 bp	CCCAGCCAAGCCCTGCACTGAT
nAChR β3 rat	145 bp	AAGGACCCCATGGACCGCTTCT
nAChR β4 rat	72 bp	CTGGTCAGGGTCCCTCATCCCAG
nAChR α2 rat	103 bp	CTCCATGGCTCCCCGGATCTGAA
nAChR α3 rat	68 bp	TTGAACCTGCTCCCCAGGGTCATG
nAChR α4 rat	356 bp	TGGGTGAAGCAGGAGTGGCACGA
nAChR α5 rat	77 bp	TGTCTTTGCCATCAACATCCACCACC
nAChR α7 rat	214 bp	CAAGAGCTCCTGCTACATTGACGTTCGC
18S rRNA rat	133 bp	TGGAGCGATTTGTCTGGTTAATTCCGATAAC

**Table 2:** Oligo sequences of primers.

Real-time PCR primers	+ Primer	- Primer
nAChR β2 rat	CTGCGGCTGACCCATGTAC	TGGGCTCAGCTCGGAAAG
nAChR β3 rat	CCCGAGATGGCTTTGCAT	GGAAAGCGACCAGAACTCTTTC
nAChR β4 rat	CGTCCCGGTCTTGAAGTCA	CAGTATCAGCTGTGGCCAAGTG
nAChR α2 rat	TGCCCAGGTGGCTGATG	CATGTTAGTCTCTAGCCAATGGTATGA
nAChR α3 rat	TTGGGTCAAGGCCGTGTT	TCACCACTGGTCGGCCTAGT
nAChR α4 rat	CCAGATGATGACAACCAACG	CCACACGGCTATGAATGCTC
nAChR α5 rat	GATTTTCGTGACCCTATCCATTATG	GCGTTGTGTGTGGAGGAAGA
nAChR α7 rat	TTGCCAGTATCTCCCTCCAG	CTTCTCATTCCTTTTGCCAG
18S rRNA rat	GTGCATGGCCGTTCTTAGTTG	GCCACTTGTCCCTCTAAGAAGTTG

### RT reaction

A cDNA library representing each CA1 hippocampal interneuron was made by adding the aspirated cytoplasm (see Cytoplasm Aspiration section) to a reverse transcription reaction using BIORAD iScript cDNA Synthesis Kit with a final volume of 10 μl.

### Multiplex reaction and real-time quantitative PCR

A multiplex PCR reaction was run (15 cycles) for each aspirated interneuron using all neuronal nAChR primers and the primer for 18S rRNA (see Tables 1 and 2) with a final volume of 75 μl. The α6 subunit was not examined because initial experiments showed no detection of this subunit in any hippocampal interneurons examined. The multiplex reaction was run using Platinum^®^ *Taq* DNA Polymerase and PCR nucleotides (10mM). A second round of PCR was run (60 cycles) for each specific target (18S, α2–α5, α7, β2, β3, β4) using an ABI 7000 Sequence Detection System utilizing BIORAD iTaq Supermix with ROX. Cycle threshold values for each target were compared to the reference gene 18S for analysis (more in Real-Time Analysis).

Standard curves (efficiency tests) for each cDNA target were developed by running 60-cycle real-time quantitative PCR assays on positive controls (rat whole-brain homogenate) for six known concentrations (100, 33.3, 10, 3.33, 1, 0.333 ng cDNA/μL). Two types of negative controls were run as well. First, we aspirated extracellular fluid and tested for the presence of nAChR mRNA. Any subunit that had a cycle threshold greater than the mRNA detected in the extracellular fluid was omitted from that interneuron’s profile. The second negative control was to simply run the quantitative PCR assay with no cellular components. This was used to detect any mRNA contaminants.

To correct the standard curves for efficiency so all mRNAs were equally efficient, upstream (primer +) and downstream (primer–) primer concentrations were adjusted to optimize amplification. The efficiency of the amplification reaction is calculated using the slope of the log(concentration) vs. CT plot. The formula for PCR efficiency = 10^(−1/*slope*)^ − 1 [23]. Reaction efficiencies were run in triplicate and the amplification efficiencies were compared using an ANOVA to determine if there were significant differences between any of the primer/probe sets (18S, α2–α5, α7, β2, β3, β4).

To calculate primer efficiencies, triplicate reactions of each cDNA target were averaged and a linear regression equation was calculated (SLOPE function, Microsoft Excel) of the CT values corresponding to the six known concentrations (100, 33.3, 10, 3.33, 1, 0.333 ng cDNA/μL) in the standard curve primer efficiency tests. The PCR efficiency was then determined by incorporating the slope of the linear equation using the formula described above.

### Data and statistical analysis

Following the real-time quantitative PCR on hippocampal interneurons, raw fluorescence (ΔRn) values across 60 cycles were curve-fit using a Boltzmann Sigmoidal function with an output of either 2000 or 4000 data points in the new curve using GraphPad v. 4.0 software. The second derivative graph for the curve-fit data was then determined, also using Graphpad. The cycle threshold (CT) value used was the maximum second derivative value of the fluorescence from the curve fit.

For comparison of expression levels between cDNA targets, fold expression values from the triplicate CT averages were calculated as compared to the lowest level of cDNA detection using the 2^-ΔΔCt^ method described by [24]. Significance between relative levels of mRNA expression was calculated by comparing mean fold expression values using a Mann-Whitney test (calculated using InStat ver. 3.05).

For the RT-PCR analysis, the proportion of neurons expressing a particular mRNA is equal to the number of observed positive neurons divided by the total number of neurons analyzed for that mRNA transcript. The standard error of the proportion (S.E.P.) was calculated for each mRNA transcript examined using the following formula [25]:

$$S.E.P.\;=\left(\frac{p\left(1-p\right)}{n}\right)$$ (1)

where *p* = the proportion of neurons in the population with detectable expression, and *n* = the number of neurons in that population.

To test for significant expression of each mRNA when compared to the background RT-PCR calculated false positive proportion, and to compare the mRNA expression of these two neuronal populations which have previously been shown to have functional nAChR responses, we used a *z*-test for comparing two sample proportions. The formula is [26]:

$$z=\frac{{\hat{p}}_{1}-{\hat{p}}_{2}}{\sqrt{\hat{p}(1-\hat{p})\left(\frac{1}{n_{1}}+\frac{1}{n2}\right)}}$$ (2)

where ${\hat{p}}_{\text{x}}$ is the proportion of cells with detected expression in each population, n_x_ is the number of neurons examined in that population, and $\hat{p}$ (no subscript) is the pooled estimate of ${\hat{p}}_{1}$ and ${\hat{p}}_{2}$. The calculation for $\hat{p}$ is [26]:

$$\hat{p}=\frac{Total\;count\;of\;successes\;in\;both\;samples\;combined}{Total\;count\;of\;observations\;in\;both\;samples\;combined}$$ (3)

Each *z*-value was compared to a normal curve using the Excel NORMSDIST(absolute value of *z*) function, which gives the probability (*p*) that the two proportions are equal. The proportions were considered significantly different when *p*≤0.05.

### Materials

Artificial cerebrospinal fluid: 124 NaCl, 2 KCl, 1 NaH_2_PO_4_, 26 NaHCO_3_, 11 Glucose, 2 CaCl_2_, 1 MgSO_4_ (in mM): Sigma-Aldrich, 3050 Spruce Street, St. Louis, MO 63103 USA.

All primers, all probes, Vector NTI version 7.0, Platinum^®^ *Taq* DNA Polymerase, PCR nucleotides: Invitrogen/Thermo Fisher Scientific, 168 Third Avenue, Waltham, MA USA 02451 USA.

Vibratome 1000-Plus: Pelco, Ted Pella, Inc., P.O. Box 492477, Redding, CA 96049–2477 USA.

Nikon E600-FN Microscope: A.G. Heinz, 20291 Valencia Circle, Lake Forest, CA 92630–8155 USA.

Borosilicate capillaries: Harvard Apparatus, 84 October Hill Road, Holliston, Massachusetts 01746 USA.

Intracellular Fluid: 10 MgCl_2_, 0.1 CaCl_2_, 1 EGTA, 10 HEPES, 135 K-Gluconate, and 2 Na-ATP (in mM): Sigma-Aldrich, 3050 Spruce Street, St. Louis, MO 63103 USA.

Multiclamp 700A: Axon Instruments/Molecular Devices, 1311 Orleans Drive, Sunnyvale, CA 94089 USA.

ABI 7000 Sequence Detection System and Primer Express version 2.0, ABI Prism: Applied Biosystems, 850 Lincoln Centre Drive, Foster City, CA 94404 USA.

iScript cDNA Synthesis Kit and iTaq Supermix with ROX: BIORAD, 2000 Alfred Nobel Drive, Hercules, California 94547 USA.

GraphPad v. 4.0 and GraphPad Instat v. 3.05: GraphPad Software, Inc., 7825 Fay Avenue, Suite 230, La Jolla, CA 92037 USA.

Microsoft Excel: Microsoft Building 92, 15010 NE 36th St, WA 98052–6399 USA.

## Results

Pregnant female Wistar rats (Wistar IGS strain code 003) were obtained from Charles River Labs. 8 to 35 days old male and female rat pups were used to make the coronal hippocampal brain slices in these experiments. The average age was 17 days old (S.E.M. 0.6 days). A total of 93 neurons were examined with reverse-transcription quantitative PCR (RT-qPCR). 42 of the neurons were from the CA1 *stratum oriens*, and 51 were from the *stratum radiatum* (see S1 Fig). Our results highlight the heterogeneity of nAChR mRNAs in hippocampal interneurons. This is in agreement with the variety of kinetics observed using whole-cell electrophysiology in response to ACh applications (Fig 1). This kinetic diversity indicates a need for greater differentiation of nAChR subtypes. Real-time quantitative PCR (qPCR) of individual interneurons of the *stratum oriens* and *stratum radiatum* showed that most (83 of 93 cells, 89.2%) interneurons expressed detectable mRNA levels of at least 1 nAChR subunit. Fig 2 shows the number of nAChR subunits (as mRNA) that were detected per neuron. The overall average number of different nAChR subunit mRNAs detected per neuron was 3.4 (± 0.2 S.E.M.), with a maximum of 8 (found in 2 cells).

To identify mRNA co-expression patterns, we calculated the most commonly co-expressed pairs of subunits (Table 3). The two subunits that co-express at the highest rate in this sample are the α3 and β2, where they were seen in just over half of the interneurons in this population (52%, n = 69 for cells expressing at least 2 subunits). This analysis was done qualitatively (i.e., a cell is either not expressing or is expressing a particular subunit: 0 or 1). This qualitative analysis has the advantage of simplifying the analysis for the co-detection of subunit pairs (or triplets, quadruplets, etc.), and also allows for the detection of combinations where the actual levels of expression might be very different.

**Table 3:** Subunit co-expression of subunit mRNA.

Percentage^1^	Subunit Co-expression
52%	α3 and β2
49%	α3 and α5
44%	α7 and β2
42%	α5 and β2
41%	α3 and β4
41%	β2 and β4
39%	α3 and β3
38%	β2 and β3

^1^ The percentage of subunit co-expression for the highest 8 subunit pairs (n = 69, the number of cells that contained at least 2 mRNA subunits). For example, 36 interneurons expressed both the α3 and β2 subunit mRNAs (36/69*100 = 52%).

**Fig 4:**
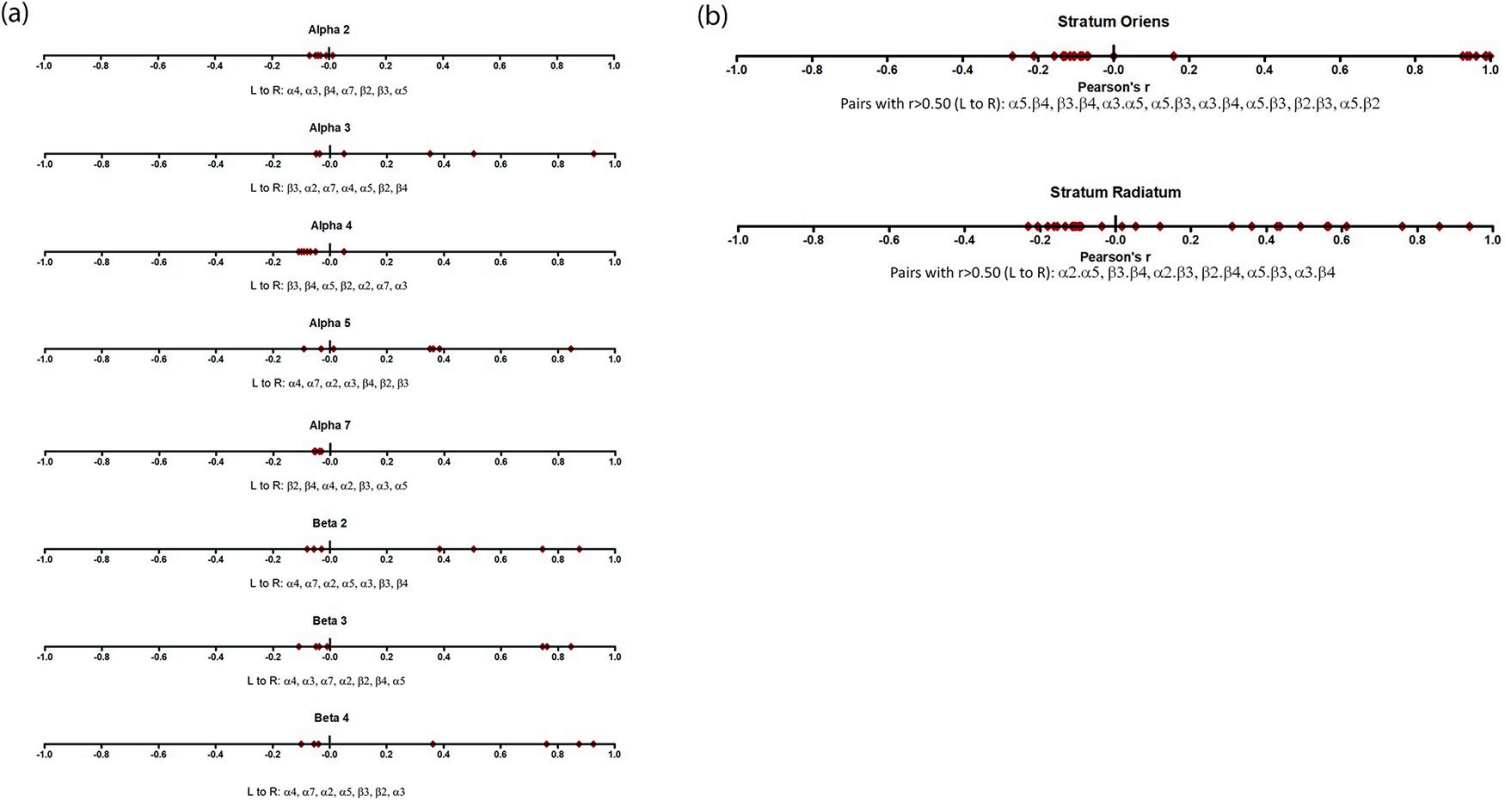
Pearson r correlations of the average fold expression for 2-way comparisons. Correlation coefficients (Pearson r values) of the quantitative expression levels of nAChR subunit mRNAs. Panel A shows the combined data from both the *stratum oriens* and *stratum radiatum*, listed by subunit. Panel B shows the correlations divided by hippocampal layer: *stratum oriens* and *stratum radiatum*. The *stratum radiatum* has a greater variability in Pearson r values while the *stratum oriens* has Pearson r values centered near zero and a separate group with values greater than 0.90. Noted correlations (those with r > 0.5) are listed from smallest to largest (left to right).

To also take advantage of the quantitative measurements of subunit mRNA expression to look for potential patterns of subunit co-expression, we calculated correlations in the actual amount of co-expressed subunits using the Pearson r correlation values for fold expression of subunits within neurons. Fig 4A shows the ranges of Pearson r values for the co-expression of each subunit compared to all of the other subunits. Fig 4A shows the pooled correlations for interneurons regardless of location in the *stratum oriens* or *stratum radiatum*. These results show that the levels of expression of all the β mRNA subunits are highly correlated with each other, with r values between 0.7 and 1. Also, both the α3 and α5 levels of expression appear to be correlated with the β2 and β4 mRNA subunits. The Pearson r values for the α2, α4, and α7 subunits are all close to zero, indicating that in the pooled sample, the mRNA levels of expression for these subunits do not appear to be highly correlated with any other particular subunits.

To see if there was any evidence for co-expression differences in different hippocampal layers, we divided the cells by hippocampal locations (Fig 4B), and re-computed Pearson r values for mRNA co-expression levels. The *stratum radiatum* results show a wide range in correlation values. The overall range is similar to the *stratum oriens*; however, the *stratum oriens* appears to consist of two discrete groupings: those not correlated, with r values close to zero, and those with very strong correlations, grouped to the right in Fig 4B with r values > 0.9. Therefore, we tested the Pearson r correlation values to see if they were significantly different across the *stratum oriens* and *stratum radiatum* [27]. Table 4 summarizes our results. To focus on highly correlated subunit pairs, we only tested those with r values greater than 0.9 in either the *stratum oriens* or *stratum radiatum*. All of the subunit pairs listed in Table 4 show significantly different correlation coefficients between the *stratum radiatum* and the *stratum oriens* (p-value<0.05, two-tailed). This data indicates that the single-cell subunit co-expression profiles are significantly different between the *stratum oriens* and the *stratum radiatum*, a likely indication that there are some different nAChR subtypes expressed in the two areas.

**Table 4:** Comparison of Pearson correlation coefficients.

subunit	subunit	*Stratum oriens*	*Stratum radiatum*	p-value^1^
α3	α5	0.961	0.361	<0.0001
α3	β4	0.987	0.937	0.00527
α5	β2	0.996	0.053	<0.0001
α5	β3	0.986	0.858	<0.0001
α5	β4	0.926	0.118	<0.0001
β2	β3	0.989	0.428	<0.0001
β2	β4	0.944	0.759	0.00829
β3	β4	0.938	0.565	0.00022

^1^ Test of the Pearson r correlation coefficients for subunit co-expression in the stratum oriens vs. the stratum radiatum. The selected comparisons were done because at least one location (oriens or radiatum) had a Pearson correlation coefficient >0.90. All regions analyzed showed significance at the 0.05 level using a two-tailed t-test [27].

In addition to 2-way subunit combinations, we also analyzed all of the 3-way, 4-way, 5-way, 6-way, 7-way, and 8-way combinations that were observed (Table 5). The “expected values” were calculated by multiplying the individual expression proportions detected for each subunit in the combination (values from Table 6). As an example, the expected proportion of neurons co-expressing α2 + α3 would be 0.37 X 0.54 = 0.2, or 20%. The actual proportion of cells observed with both α2 + α3 was 0.23. So the Observed/Expected value is 1.1, very close to what would be expected, and not significantly different (p = 0.31). A *z*-test of two proportions (see Eq 2 in Data and Statistical Analysis in the Methods section) indicated that 63 different combinations appeared more often than expected. Significance values were calculated as described in the methods and are listed in Table 5.

**Table 5:** mRNA combinations with higher than expected expression (p≤0.05).

Combination	Observed/Expected ^1^	Combination	Observed/Expected ^1^
α3+α5	1.6 (p = 0.02)	α4+α5+α7+β2	4.1 (p = 0.01)
α3+α4+α7	2.7 (p = 0.01)	α4+α7+β2+β3	4.5 (p = 0.01)
α3+α5+β2	2.2 (p = 0.004)	α4+α7+β2+β4	3.4 (p = 0.04)
α3+α5+β3	2.3 (p = 0.01)	α4+α7+β3+β4	4.0 (p = 0.03)
α3+α5+β4	2.3 (p = 0.008)	α4+β2+β3+β4	3.1 (p = 0.05)
α3+β2+β3	2.1 (p = 0.01)	α5+α7+β2+β3	3.3 (p = 0.01)
α3+β2+β4	2.0 (p = 0.01)	α5+α7+β2+β4	2.6 (p = 0.04)
α3+β3+β4	2.4 (p = 0.009)	α5+α7+β3+β4	3.2 (p = 0.03)
α4+α7+β2	3.0 (p = 0.005)	α5+β2+β3+β4	3.9 (p = 0.005)
β2+β3+β4	2.3 (p = 0.01)	α7+β2+β3+β4	3.4 (p = 0.01)
α2+α3+α5+β3	2.9 (p = 0.04)	α2+α3+α7+β2+β3	4.9 (p = 0.02)
α2+α3+α7+β2	2.4 (p = 0.05)	α3+α4+α5+α7+β2	7.7 (p = 0.004)
α2+α3+β2+β3	2.6 (p = 0.04)	α3+α4+α7+β2+β3	6.6 (p = 0.01)
α2+α4+α7+β2	3.9 (p = 0.03)	α3+α4+α7+β2+β4	6.3 (p = 0.01)
α2+α7+β2+β3	3.0 (p = 0.04)	α3+α4+α7+β3+β4	7.4 (p = 0.01)
α3+α4+α5+α7	4.1 (p = 0.01)	α3+α4+β2+β3+β4	5.8 (p = 0.02)
α3+α4+α5+β2	3.9 (p = 0.01)	α3+α5+α7+β2+β3	5.7 (p = 0.004)
α3+α4+α7+β2	4.6 (p = 0.003)	α3+α5+α7+β2+β4	4.4 (p = 0.02)
α3+α4+α7+β3	3.6 (p = 0.03)	α3+α5+α7+β3+β4	6.0 (p = 0.01)
α3+α4+α7+β4	3.4 (p = 0.04)	α3+α5+β2+β3+β4	7.3 (p = 0.0008)
α3+α4+β2+β3	2.8 (p = 0.05)	α3+α7+β2+β3+β4	6.4 (p = 0.002)
α3+α4+β2+β4	3.3 (p = 0.02)	α4+α7+β2+β3+β4	7.4 (p = 0.01)
α3+α4+β3+β4	3.6 (p = 0.03)	α5+α7+β2+β3+β4	6.0 (p = 0.01)
α3+α5+α7+β2	3.3 (p = 0.005)	α2+α3+α5+α7+β2+β3	8.5 (p = 0.02)
α3+α5+α7+β3	3.1 (p = 0.02)	α2+α3+α7+β2+β3+β4	8.7 (p = 0.02)
α3+α5+β2+β3	3.5 (p = 0.004)	α3+α4+α5+α7+β2+β3	11.6 (p = 0.01)
α3+α5+β2+β4	3.5 (p = 0.003)	α3+α4+α5+α7+β3+β4	12.4 (p = 0.02)
α3+α5+β3+β4	4.5 (p = 0.002)	α3+α4+α7+β2+β3+β4	13.8 (p = 0.01)
α3+α7+β2+β3	3.3 (p = 0.01)	α3+α5+α7+β2+β3+β4	11.1 (p = 0.004)
α3+α7+β2+β4	2.7 (p = 0.02)	α4+α5+α7+β2+β3+β4	12.4 (p = 0.02)
α3+α7+β3+β4	3.4 (p = 0.01)	α3+α4+α5+α7+β2+β3+β4	23.0 (p = 0.02)
α3+β2+β3+β4	4.0 (p = 0.001)		

^1^ Expected values were calculated by multiplying the proportion values from Table 6. Observed values were a count of the occurring combination.

**Table 6:** Proportion of cells by subunit.

Subunit	Proportion^1^
α3	0.54
β2	0.54
α5	0.43
α7	0.42
β4	0.42
β3	0.40
α2	0.36
α4	0.27

^1^ Proportions were calculated by dividing the number of cells containing the specific subunit by n = 93. As a note, ten of the 93 interneurons tested did not contain detectable levels of any nAChR mRNA subunit analyzed.

Table 7 lists all of the mRNA subunit combinations we actually observed, as well as their location in either the *stratum oriens* or *stratum radiatum*. Interestingly, the two most commonly observed combinations were fairly complex: α3α4α5α7β2 and α3α5β2β3β4. The former occurred 4 times solely in the *stratum radiatum* while the later appear 2 times in the *stratum radiatum* and 2 times in the *stratum oriens* (for a total of 4). Our analysis revealed 59 different combinations of mRNA subunit expression in the 93 cells tested. Of the 93 cells, 10 cells did not have detectable levels of expression and 42 cells had a unique combination of mRNA subunits.

**Table 7:** All mRNA subunit combinations observed by location.

Combination	Number of Occurrences (Radiatum)	Number of Occurrences (Oriens)	Combination	Number of Occurrences (Radiatum)	Number of Occurrences (Oriens)
No nAChR mRNA	4	6	α2α4α7β2	-	1
α2	2	-	α2β2β3β4	1	-
α3	-	1	α3α4β2β4	1	-
α4	1	-	α3α5β2β4	-	2
α5	1	1	α3α7β2β3	-	1
α7	1	1	α3β2β3β4	-	1
β2	2	1	α4α5β2β4	-	1
β3	2	1	α4α7β2β3	1	-
α2α3	2	-	α5α7β2β3	1	-
α2α7	-	1	α5α7β2β4	1	-
α2β2	1	-	α2α3α4α5β 2	1	-
α2β4	2	-	α2α3α4α7β2	1	-
α3β4	-	1	α2α3α5β3β4	-	1
α4α7	1	-	α2α4α7β2β3	1	-
α5β4	1	-	α3α4α5α7β2	4	-
α7β3	1	-	α3α4α5β2β4	-	1
α7β4	1	-	α3α4α5β3β4	1	-
α2α3α5	1	-	α3α5β2β3β4	2	2
α2α3β2	-	1	α2α3α4α7β2β4	-	1
α2α7β2	-	1	α2α3α5α7β2β3	1	-
α2β3β4	1	1	α2α3α5β2β3β4	1	-
α3α4α7	-	1	α2α3α7β2β3β4	-	1
α3α5α7	2	-	α3α4α7β2β3β4	-	1
α3α5β2	1	-	α3α5α7β2β3β4	2	-
α3α5β3	1	-	α2α3α4α5α7β2β3	1	-
α3α7β3	-	1	α2α3α4α7β2β3β4	-	1
α7β2β4	1	1	α2α3α5α7β2β3β4	-	2
α2α3α5β3	1	-	α3α4α5α7β2β3β4	-	3
α2α3α5β4	-	2	α2α3α4α5α7β2β3β4	1	1
α2α3β2β3	1	-			

To estimate how well our sample of cells represented the actual underlying diversity of nAChR subunit expression patterns in hippocampal interneurons we used the coverage estimation calculation provided by [28]. This calculation estimated that our sample, as diverse as it was, actually only had 49.4% coverage–implying that we only observed about half of the underlying subunit combinations expressed in these neurons.


$$\begin{array}{ll} \text{Estimated}\;\text{sample}\;\text{coverage} & =1-\left(\#\;\text{single}\;\text{observations}/\#\;\text{of}\;\text{total}\;\text{observations}\right)\\ & =1-\left(42/83\right)\\ & =0.494 \end{array}$$

Chao and Lee [28] also provide a way to calculate the estimated number of possible combinations.


$$\begin{array}{l} =\left(\#\;\text{of}\;\text{combinations}\;\text{observed}\right)/\text{coverage}\\ =59/0.494\\ =119.4 \end{array}$$

This calculation gives an estimate of over 119 different nAChR subunit mRNA combinations that could be found in the inhibitory interneurons of the CA1 of the rat hippocampus.

## Discussion

Our results indicate that while there may be similarities in the nAChR subunit mRNA expression between the *stratum oriens* and the *stratum radiatum* there are also some significant differences. One important observation is the high rate of expression of the α3 subunit mRNA in hippocampal interneurons. Another very important finding is the sheer overall diversity of subunit combinations detected, with an estimated 119 different underlying combinations of nAChR subunit mRNA expression.

It would be interesting to further explore changes in subunit combinations during development [29–32]. The role of nAChRs in development may be quite diverse considering that nAChRs are found on glial cells, pyramidal cells, and neurons [33]. A better understanding of nAChR subtypes, and how they change with aging, could improve treatment of ADHD or early intervention therapies for ASD. Likewise, understanding normal aging as opposed to dementia or Alzheimer’s Disease may improve targeted therapies, especially since some nAChR subtypes have been shown to be sensitive to β-amyloid [34, 35].

Another interesting result is that in those interneurons that expressed only a single subunit, some of those interneurons expressed the α7 mRNA. Since the α7 subunit can form a homomer those results are expected. However, there were also some interneurons with the sole detection of the α2, α3, α4, α5, β2, β3, or the β4 mRNA subunits, even though none of these subunits can form homomeric channels. The results are likely the result of taking a snapshot view of mRNA levels at any one time in the interneuron. It is likely that nAChR mRNA levels within a neuron rise and fall in response to developmental cues and cellular needs, and we likely missed the detection of other subunits that would be expressed at different times.

The high levels of co-expression of α3 and β2 (Table 3) were somewhat surprising because the α3 has only been shown to be primarily in the peripheral nervous system (PNS) in the autonomic ganglia, and very sparse in the CNS–with the exception of the medial habenula [36]. However, our results suggest that that the α3 subunit has a significant role in hippocampal interneurons. Since the α3 and β2 subunits had the highest overall expression and the highest co-expression rate, it highlights the importance of further characterizing the α3β2 nAChR.

Our results also indicate that α3 containing cells frequently expressed α5 mRNA as well. We suggest that the α3β2, and perhaps α3α5β2 nAChRs, may play a more important role in the hippocampus than the α4β2 nAChRs. Not as surprising were the relatively abundant α7 mRNA expressing cells. α7 appears to be fairly average in relative expression levels, with 42% of neurons with detectable expression (Table 6) and the fold expression mainly between 0.1X to 10X the median (Fig 3). This validates the potential use of α7 targeted therapies in the hippocampus–with the caveat that they would not provide for specific targeting to the hippocampus, since α7 receptors are found in many locations in the CNS. Considering that α3 is found in very few regions of the brain and α7 has a more ubiquitous expression, α3 targeted therapies may prove effective [36–39], but only if they can be targeted to α3β2 receptors to avoid the α3β4 receptors that are highly expressed in autonomic ganglia in the peripheral nervous system.

Future studies may include reconstituting the significant combinations of nAChR subunit co-expression (Table 3) into cellular expression systems such as *Xenopus laevis* oocytes. Studies using expression systems benefit from the finer control of diffusion rates and drug application concentrations versus interneurons within the brain slice. Findings from electrophysiological recordings in response to pharmacological application can then be used to elucidate the characteristics and contributions of the significant nAChR subunit co-expression combinations identified in this study (Table 3). These studies would then help us understand the role of nAChRs in modulating hippocampal function. Further pursuing these results may identify networks or subpopulations in the hippocampus and throughout the CNS affected in the pathogenesis of neurodegenerative diseases and ultimately give rise to the discovery of treatments that prevent or abate these disorders. The results in this study are a gateway to the characterization of the diverse range of hippocampal nicotinic receptor subtypes.

## Supporting information

S1 Fig Data for all neurons examined. The original hippocampal layer location of aspirated neurons (stratum oriens vs. stratum radiatum), the ages of rats the samples were taken from (average age and S.E.M. are shown at the bottom), and the deltaCT values from the quantitative RT-PCR analysis for nAChR subunit mRNA. The column on the right side shows the number of subunits observed from each neuron (average and S.E.M. are shown at the bottom). The bottom of the table shown the number of cells and percentage of cells expressing each subunit mRNA.
(TIF)
