# Supplementary figures and images for: Single-cell quantitative expression of nicotinic acetylcholine receptor mRNA in rat hippocampal interneurons
Source: PLoS One. 2024 Apr 18;19(4):e0301592. doi: 10.1371/journal.pone.0301592 (PMC11025973; doi:10.1371/journal.pone.0301592)

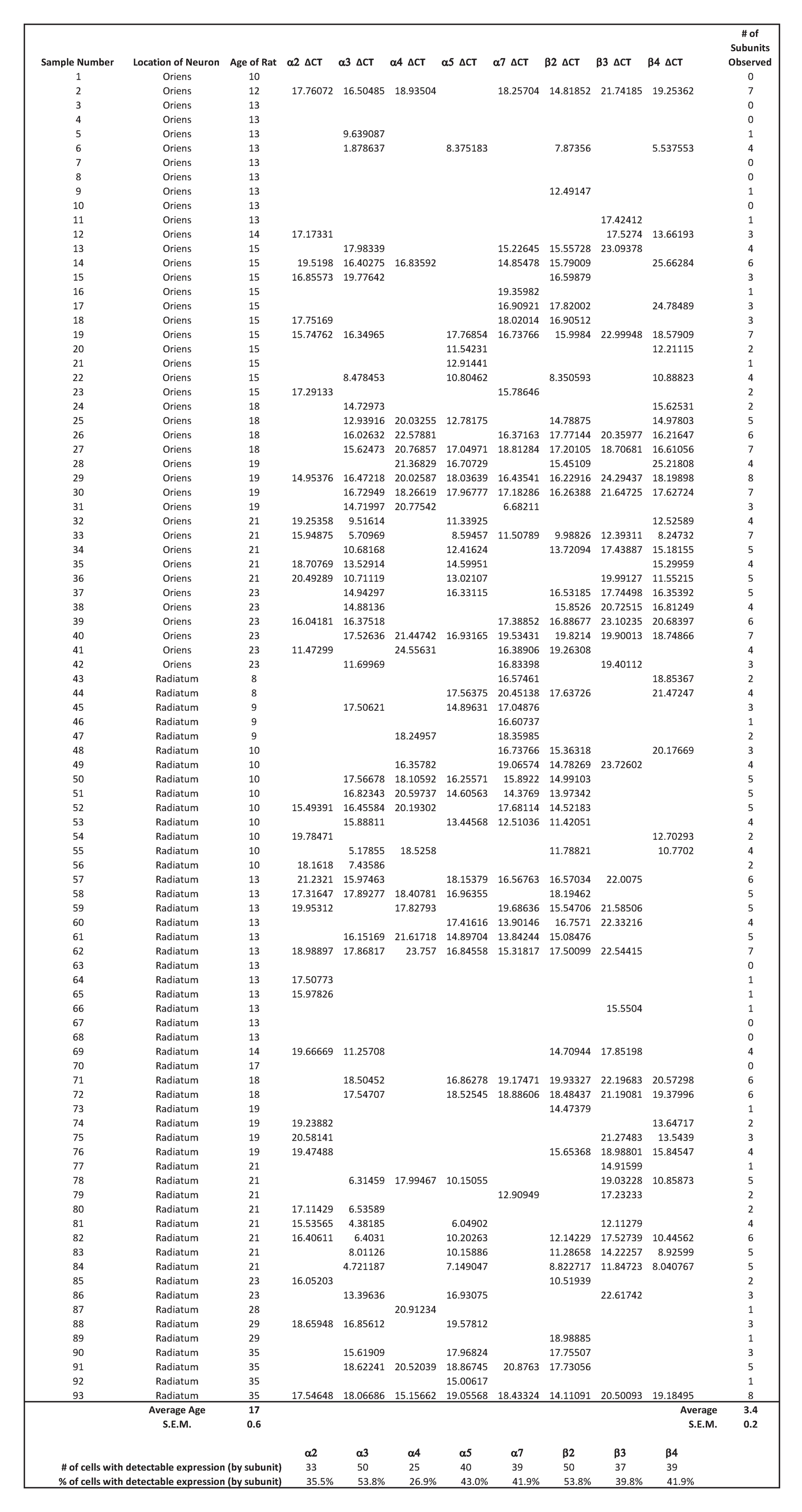

Supplement: S1 Fig — The original hippocampal layer location of aspirated neurons (stratum oriens vs. stratum radiatum), the ages of rats the samples were taken from (average age and S.E.M. are shown at the bottom), and the deltaCT values from the quantitative RT-PCR analysis for nAChR subunit mRNA. The column on the right side shows the number of subunits observed from each neuron (average and S.E.M. are shown at the bottom). The bottom of the table shown the number of cells and percentage of cells expressing each subunit mRNA. (TIF) [file pone.0301592.s001.tif]
